# First records of *Gnathia* Leach, 1814 and *Tachaea* Schioedte & Meinert, 1879 from South Korea, with descriptions of two new species (Isopoda, Cymothoida, Cymothooidea)

**DOI:** 10.3897/zookeys.787.26291

**Published:** 2018-10-02

**Authors:** Ji-Hun Song, Gi-Sik Min

**Affiliations:** 1 Department of Biological Sciences, Inha University, 100 Inha-ro, Nam-gu, Incheon 22212, South Korea Inha University Incheon Korea, South

**Keywords:** Corallanidae, *
Gnathia
*, Gnathiidae, Isopoda, South Korea, *
Tachaea
*

## Abstract

Two new species of cymothoid isopods, *Gnathiakoreana***sp. n.** and *Tachaeakoreaensis***sp. n.**, are described from South Korea. The genera *Gnathia* Leach, 1814 and *Tachaea* Schioedte & Meinert, 1879 are recorded for the first time in South Korea. *Gnathiakoreana***sp. n.** is distinguished from its congeners by having the smooth dorsal surface of the pereon, the strongly ridged unornamented paraocular ornamentation, the strong bifid mediofrontal process, and the serrated superior frontolateral process. *Tachaeakoreaensis***sp. n.** is distinguished from its congeners by having the expanded propodus with serrated inferior margins in pereopods 1–3, the propodus with serrated inferodistal margins in pereopods 4–7, one seta on the apical lobe of the maxilla, and ten robust setae on the posterior margin of the pleotelson.

## Introduction

The isopod crustacean family Gnathiidae Leach, 1814 is one of the nine families belonging to the superfamily Cymothooidea Leach, 1814. This family is unusual among isopods as its members exhibit peculiar morphological differences between juveniles (praniza stage) and adults (Cohen and Poore 1994, Ota 2012, Tanaka 2004, Tanaka and Nishi 2011). In addition, they have biphasic life cycles with ectoparasitic larva (praniza stage) and free-living adults (Hadfield et al. 2009, Ota et al. 2012, Smit et al. 2003). Pranizas are regarded to be parasites of fishes, whereas adults are non-feeding and have a degenerated intestine (Golovan 2006; Tanaka and Nishi 2011). The genus *Gnathia* Leach, 1814 is the largest group in this family and is currently composed of 126 species distributed worldwide (Boyko et al. 2008; Cohen and Poore 1994).

The family Corallanidae Hansen, 1890 also belongs to the superfamily Cymothooidea. The genus *Tachaea* Schioedte & Meinert, 1879 is the smallest group in this family and is currently composed of seven species (Boyko et al. 2008). The type species, *Tachaeacrassipes* Schioedte & Meinert, 1879 is the only marine species found in the coral reefs of Singapore, whereas other species are found in freshwater habitats as ectoparasites of fish and various shrimp species (Delaney 1989).

Herein, we report two new species collected from South Korea, *Gnathiakoreana* sp. n. and *Tachaeakoreaensis* sp. n. The genera *Gnathia* and *Tachaea* were first found in the United Kingdom and Singapore, respectively, but the present study represents the first record of these genera in South Korea.

## Materials and methods

### Sampling

Specimens of *G.koreana* sp. n. were collected using light traps from Geomun-do Island (approximately 10 m depth) in South Korea. The sediment at the sampling site was characterized as organic-rich muddy sand. Specimens of *T.koreaensis* sp. n. were collected as ectoparasites on the freshwater shrimps *Macrobrachiumnipponense* (De Haan, 1849) and *Palaemonpaucidens* De Haan, 1844 collected from reservoirs in South Korea. All specimens were preserved immediately after collection in 95% ethyl alcohol. The type specimens of the two new species have been deposited in the National Institute of Biological Resources (**NIBR**), Incheon, South Korea.

### Morphological analysis

The specimens were transferred to glycerine for dissection, and then examined and dissected under a dissection microscope (Olympus, model SZX-7). Figures of dissected appendages were drawn under a light microscope with an attached drawing tube (Leica, model DM 2500). Figures of the whole body were drawn using a drawing tube attached to a stereomicroscope (Olympus, model SZX-12). The lengths of all appendages and the whole body were measured with a stage micrometre (Leica, model no. 11513106) and an ocular micrometre. The photograph of the whole body of *G.koreana* sp. n. was taken using a digital camera (eXcope, model K6) mounted on a stereomicroscope, and those of the cephalon was taken using a scanning electron microscope (Hitachi, model S-4200). Pre-treatments were performed based on the methods described by Song and Min (2016).

Morphological terminology and the orientation of each appendage largely follows Bruce (2009); some morphological terms were taken from Cohen and Poore (1994) to retain descriptive consistency for the cephalic appendages of *G.koreana* sp. n. Setal terminology largely follows Watling (1989). Unless otherwise specified, the setae are simple.

## Taxonomy

### Suborder Cymothoida Wägele, 1989

#### Superfamily Cymothooidea Leach, 1814

##### Family Gnathiidae Leach, 1814

###### Genus Gnathia

Taxon classificationAnimaliaIsopodaGnathiidae

Leach, 1814

####### Type species.

*Gnathiamaxillaris* (Montagu, 1804) by original designation.

###### Key to the species of *Gnathia* from Japan, Korea, and Russian Far East

This key is based on males.

**Table:** 

1	Dorsal surface of pereon without tubercles	**2**
–	Dorsal surface of pereon with tubercles, especially anteriorly	**13**
2	Paraocular ornamentation absent; mandible without incisor	**3**
–	Paraocular ornamentation present; mandible with incisor	**8**
3	Dorsal surface of cephalon with tubercles; pylopod with three articles	***G.limicola* Ota & Tanaka, 2007**
–	Dorsal surface of cephalon without tubercles; pylopod with two articles	**4**
4	Body very setose; inferior margins of pereopods without tubercles	***G.capillata* Nunomura & Honma, 2004**
–	Body smooth or sparsely setose; inferior margins of pereopods with tubercles	**5**
5	Mediofrontal process bifid	**6**
–	Mediofrontal process a single projection	**7**
6	Mediofrontal process broad, not elongated; superior frontolateral process triangular; mandible mediocre	***G.bungoensis* Nunomura, 1982**
–	Mediofrontal process narrow anteriorly, elongated; superior frontolateral process rounded; mandible stout	***G.mutsuensis* Nunomura, 2004**
7	Mediofrontal process acute; mandibular setae absent	***G.nasuta* Nunomura, 1992**
–	Mediofrontal process rounded; mandibular setae present	***G.sanrikuensis* Nunomura, 1998**
8	Mediofrontal process absent	***G.maculosa* Ota & Hirose, 2009**
–	Mediofrontal process present	**9**
9	Mediofrontal process dividing into two apices; internal lobe of mandible absent	**10**
–	Mediofrontal process not divided, a single projection; internal lobe of mandible present	**12**
10	Lateral margins of pereonites 2 and 3 with serrations; mediofrontal process broad, with remarkably concave apex	***G.scabra* Ota, 2012**
–	Lateral margins of pereonites 2 and 3 without serrations; mediofrontal process narrow, with bifid apex	**11**
11	Paraocular ornamentation forming a ridge; superior frontolateral process rounded, serrated	***G.koreana* sp. n.**
–	Paraocular ornamentation not forming a ridge, with three indistinct tubercles; superior frontolateral process acute, not serrated	***G.excavata* Ota, 2012**
12	Cephalon without tubercles; paraocular ornamentation forming a ridge; mediofrontal process conical, longer than superior frontolateral process	***G.camuripenis* Tanaka, 2004**
–	Cephalon with tubercles; paraocular ornamentation not forming a ridge, with several tubercles; mediofrontal process rounded, shorter than superior frontolateral process	***G.kumejimensis* Ota, 2012**
13	Mandible as long as or shorter than half-length of cephalon, with smooth blade	**14**
–	Mandible longer than half-length of cephalon, with dentate blade	**16**
14	Cephalon with distinct serrations on lateral margins; mandible as long as half-length of cephalon	***G.tuberculata* Richardson, 1909**
–	Cephalon serrated, but without distinct serrations on lateral margins; mandible shorter than half-length of cephalon	**15**
15	Epimera linguiform, visible dorsally on pleonites 1–5, directed to the sideward	***G.derzhavini* Gurjanova, 1933**
–	Epimera acute, visible dorsally on pleonites 4 and 5, directed downward	***G.schmidti* Gurjanova, 1933**
16	Body sparsely setose; pylopod with two articles	***G.teruyukiae* Ota, 2011**
–	Body very setose, covered with long setae; pylopod with three articles	**17**
17	Mediofrontal process broad, not elongated; epimera not visible dorsally on all pleonites	**18**
–	Mediofrontal process narrow anteriorly, elongated; epimera visible dorsally on pleonites 3–5	**20**
18	Mediofrontal process dividing into two apices; supraocular lobe with blunt apex	***G.rufescens* Ota, 2015**
–	Mediofrontal process not divided, rounded; supraocular lobe with dentate apex	**19**
19	Dorsal surface of pereonite 4 without tubercles; superior frontolateral process with four setae	***G.albipalpebrata* Ota, 2014**
–	Dorsal surface of pereonite 4 with tubercles; superior frontolateral process with several setae and tubercles	***G.parvirostrata* Ota, 2014**
20	Dorsolateral surface of pereonites 5 and 6 with tubercles; pleotelson with acute apex	***G.nubila* Ota & Hirose, 2009**
–	Dorsolateral surface of pereonites 5 and 6 without tubercles; pleotelson with rounded apex	***G.dejimagi* Ota, 2014**

###### Gnathia koreana sp. n.

Taxon classificationAnimaliaIsopodaGnathiidae

http://zoobank.org/BF1A7F54-5E72-41B3-9573-54D967EF55BE

**Figure 1.**
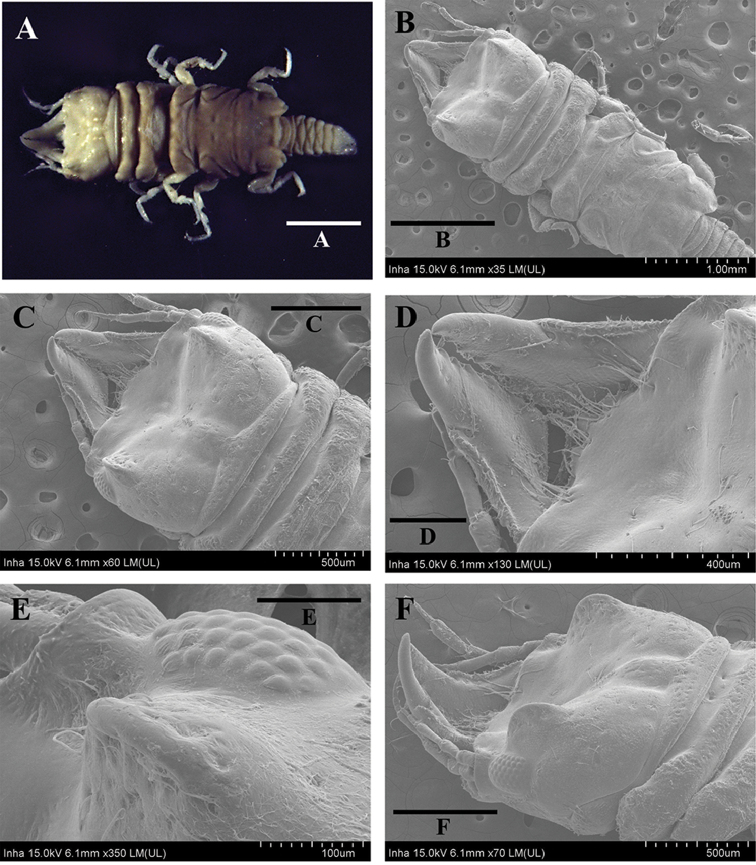
*Gnathiakoreana* sp. n., male holotype **A** body, dorsal view **B** body, dorsal view **C** cephalon **D** mandible **E** paraocular ornamentation, dorsal view **F** paraocular ornamentation, lateral view. Scale bars: 1 mm (**A, B**), 0.5 mm (**C, F**), 0.4 mm (**D**), 0.1 mm (**E**).

**Figure 2.**
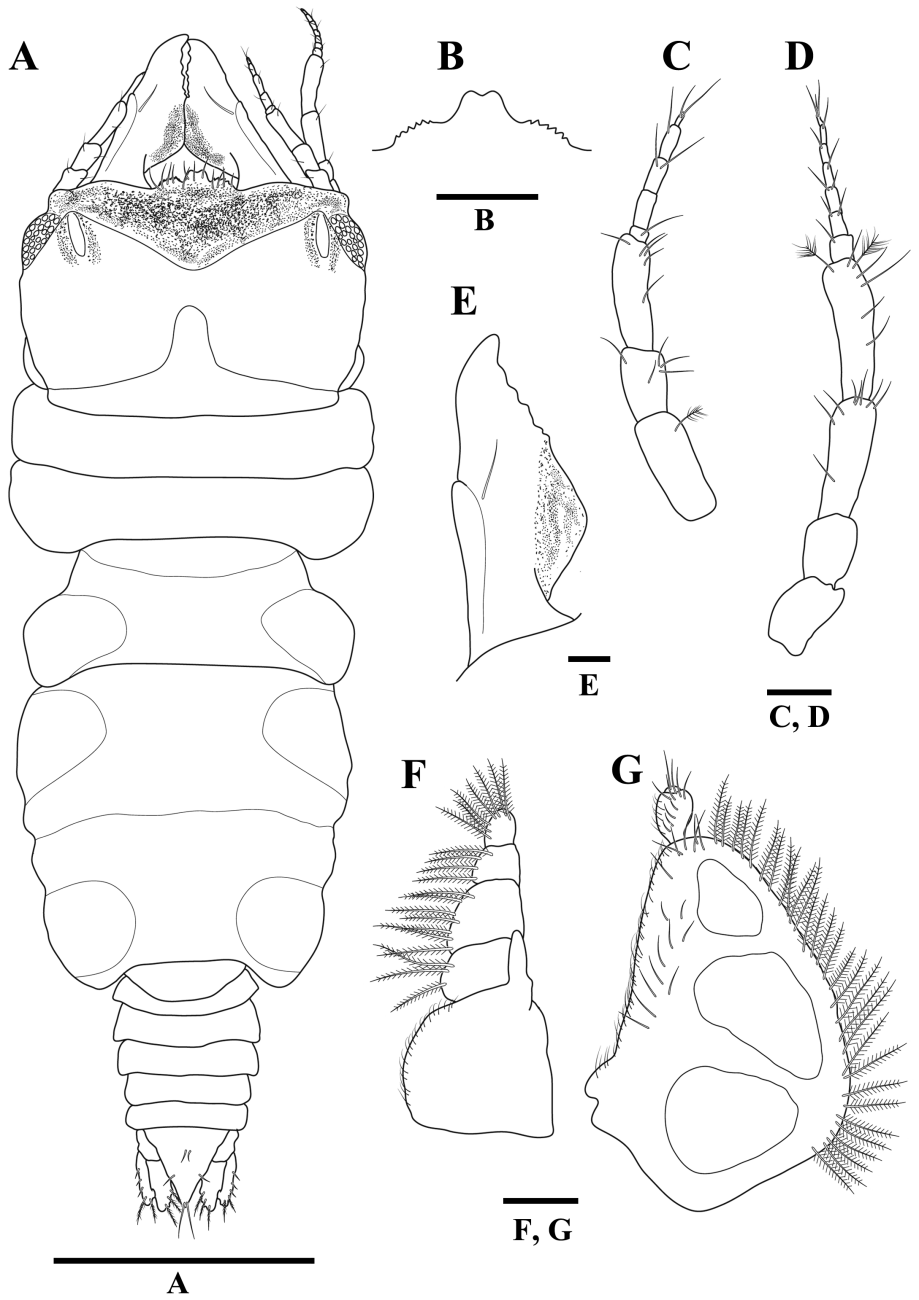
*Gnathiakoreana* sp. n., male holotype **A** body, dorsal view **B** mediofrontal process and superior frontolateral process **C** antennula **D** antenna **E** mandible **F** maxilliped **G** pylopod. Scale bars: 1 mm (**A**), 0.5 mm (**B**), 0.1 mm (**C–E**), 0.2 mm (**F, G**).

**Figure 3.**
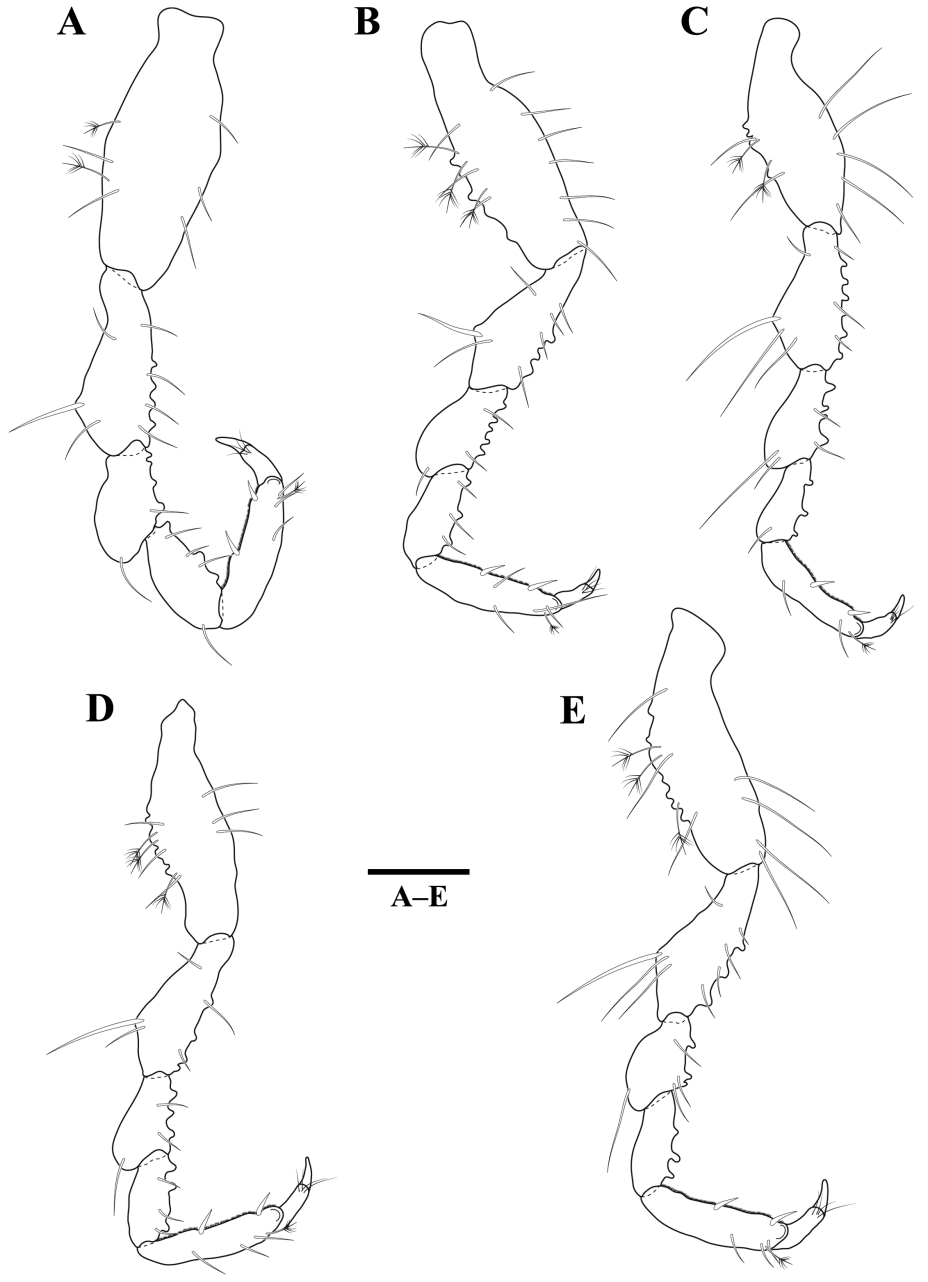
*Gnathiakoreana* sp. n., male holotype. **A** pereopod 2 **B** pereopod 3 **C** pereopod 4 **D** pereopod 5 **E** pereopod 6. Scale bars: 0.2 mm (**A–E**).

**Figure 4.**
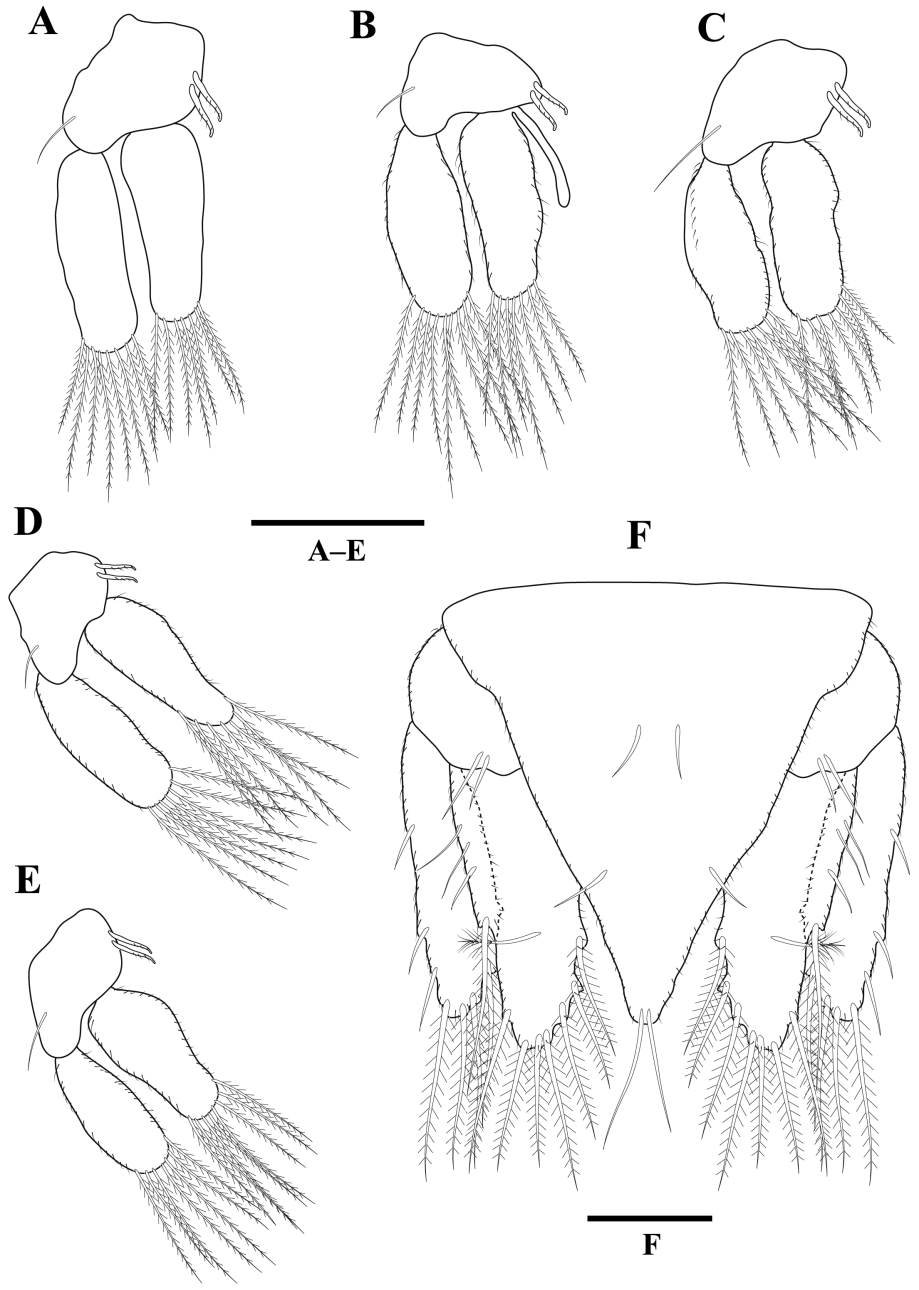
*Gnathiakoreana* sp. n., male holotype **A** pleopod 1 **B** pleopod 2 **C** pleopod 3 **D** pleopod 4 **E** pleopod 5 **F** pleotelson and uropod. Scale bars: 0.2 mm (**A–E**), 0.1 mm (**F**).

Figures 1 , 2 , 3 , 4

####### Material examined.

Holotype: adult male (4.6 mm, NIBRIV0000554213); Yeogaekseon terminal, Geomundo Island, Yeosu-si, Jeollanam-do, South Korea; 34°01'37"N, 127°18'27"E; 31 May 2014; approximately 10 m; coll. J.-H. Song. Paratype: adult male (4.3 mm, NIBRIV0000554214), same sample as holotype.

####### Etymology.

The specific name ‘*koreana*’ is derived from the name of the nation from which the specimens were collected.

####### Diagnosis.

Pereon dorsal surface smooth, sparsely setose, without tubercles. Cephalon dorsal surface sparsely setose with several granules medially. Paraocular ornamentation strongly developed, forming a ridge, without tubercles. Mediofrontal process strong and bifid. Superior frontolateral process shorter than mediofrontal process, rounded, and serrated. Mandible without pseudoblade and internal lobe.

####### Description

**(adult male, holotype).***Body* (Figures 1A, B, 2A) 3.2 times as long as greatest width; dorsal surfaces smooth, sparsely setose. *Cephalon* (Figure 1C) rectangular, 0.7 times as long as wide, lateral margins slightly convex and smooth; dorsal surface sparsely setose with several granules medially; dorsal sulcus wide, deep; paraocular ornamentation (Figure 1E, F) strongly developed, forming ridge, without tubercles; posteromedian tubercle present. *Eyes* (Figure 2A) 0.3 times as long as cephalon. *Supraocular lobe* (Figure 2A) weak, with blunt apex; accessory supraocular lobe not pronounced. *Mediofrontal process* (Figure 2B) elongated, bifid. *Superior frontolateral process* (Figure 2B) shorter than mediofrontal process, serrated. *Inferior frontolateral process* absent. *Pereonites 1–7* (Figure 2A) without tubercles on dorsal surface, lateral margins smooth; pereonite 1 not fused dorsally with cephalon, dorsolateral margins fully obscured by cephalon; pereonite 2 wider than pereonite 1; pereonite 4 with anterior constriction; areae laterales present on pereonite 5; pereonite 6 with lobi laterales; pereonite 7 narrow, overlapping pleon; lobuii weak, globular. *Pleonites 1–5* (Figure 2A), epimera not visible dorsally. *Pleotelson* (Figure 4F) 0.9 times as long as anterior width, lateral margins smooth, anterolateral margins not concave, posterolateral margins weakly concave; mid-dorsal surface with two sub-median setae, posterolateral margins with two submarginal setae, apex with two setae.

*Antennula* (Figure 2C) peduncle article 2 0.6 times as long as article 1; article 3 1.7 times as long as article 2; flagellum with five articles. *Antenna* (Figure 2D) peduncle article 4 3.0 times as long as wide, 1.2 times as long as article 3, with two penicillate setae; flagellum with seven articles.

*Mandible* (Figures 1C, D, 2E) 0.5 times as long as width of cephalon, curved upward; mandibular seta present; carina present, unornamented; incisor elevated, distally rounded; dentate blade with five weak processes; pseudoblade absent; internal lobe absent; dorsal lobe absent; basal neck short; erisma absent; lamina dentate absent. *Maxilliped* (Figure 2F) article 1 lateral margin with continuous marginal setae; article 2 lateral margin with three plumose setae; article 3 lateral margin with seven plumose setae; article 4 lateral margin with four plumose setae; article 5 with seven plumose setae; endite extending to distal margin of article 2; without coupling setae. *Pylopod* (Figure 2G) with three articles; article 1 1.4 times as long as wide, without distolateral lobe, with three areolae; posterior and lateral margins forming rounded curve; lateral margin with 30 plumose setae; mesial margin with continuous setae, distal margin with 5–6 setae; article 2 1.3 times as long as wide; article 3 minute.

*Pereopods* 2–6 (Figure 3A–E) without long plumose setae; basis superior margin with 2–3 penicillate setae; dactylus superodistal margin with one penicillate seta. Pereopod 2 basis 2.4 times as long as greatest width, superior margin with two setae, inferior margin with three setae; ischium 0.7 times as long as basis, 2.4 times as long as wide, superior margin with three setae, inferior margin with four setae and four tubercles; merus 0.6 times as long as ischium, 1.8 times as long as wide, superodistal margin with one seta, inferior margin with one seta and four tubercles; carpus as long as merus, 2.1 times as long as wide, superodistal margin with one seta, inferior margin with three setae and three tubercles; propodus 0.8 times as long as ischium, 3.5 times as long as wide, superior margin with three setae, inferior margin with two robust setae; dactylus 0.3 times as long as propodus. Pereopods 3–6 similar, but basis superior margin with 3–6 tubercles.

*Pleopods* 1–5 (Figure 4A–E) similar. Pleopod 2 exopod 2.3 times as long as wide, with 8 plumose setae; endopod 2.5 times as long as wide, with seven plumose setae; appendix masculina with parallel margins, 0.6 times as long as endopod, distally rounded. Uropod (Figure 4F) rami extending beyond pleotelson, apices rounded; peduncle with two setae; endopod 2.6 times as long as greatest width, lateral margin with three setae, mesial margin with eight plumose setae, dorsally with one penicillate seta; exopod not extending to end of endopod, 4.0 times as long as greatest width, lateral margin with three setae; mesiodistal margin with three plumose setae.

####### Habitat.

This species was collected at approximately 10 m depth corresponding to a sedimentary bottom of muddy sand.

####### Remarks.

*Gnathiakoreana* sp. n. is distinguished from other known species of *Gnathia* by the following characters: 1) the dorsal surface of the pereon without tubercles, 2) the paraocular ornamentation is strongly developed, forming a ridge, without tubercles, 3) the mediofrontal process is strong and bifid, 4) the superior frontolateral process is shorter than the mediofrontal process, rounded, and serrated, and 5) the mandible without internal lobe.

*Gnathiakoreana* sp. n. is most similar to *G.excavata* from Japan in terms of the following characters: the body is smooth, the mediofrontal process is bifid, and the mandible without internal lobe. However, the new species is distinguished from *G.excavata* by the shape of the paraocular ornamentation and superior frontolateral process. In *G.excavata*, the paraocular ornamentation with three indistinct tubercles and the superior frontolateral process is acute and not serrated. In comparison, in the new species, the paraocular ornamentation with distinct unornamented ridge and the superior frontolateral process is rounded and serrated.

####### Distribution.

Only known from the type locality.

##### Family Corallanidae Hansen, 1890

###### Genus Tachaea

Taxon classificationAnimaliaIsopodaCorallanidae

Schioedte & Meinert, 1879

####### Type species.

*Tachaeacrassipes* Schioedte & Meinert, 1879

####### Distribution.

Six species are distributed in Asia: *Tachaeachinensis* Thielemann, 1910 (China, Japan, Thailand, and Malaysia); *T.crassipes* Schioedte & Meinert, 1879 (Singapore); *T.koreaensis* sp. n. (South Korea); *T.lacustris* Weber, 1892 (Indonesia); *T.spongillicola* Stebbing, 1907 (India); and *T.tonlesapensis* Nunomura, 2006 (Cambodia). Two species are distributed in Australia: *T.caridophaga* (Riek, 1953) (Queensland); *T.picta* (Riek, 1967) (Queensland and New South Wales) (Delaney 1989; Nunomura 2006).

###### Key to the species of *Tachaea*

This key is based on females. Therefore, we excluded *T.crassipes* that is designated the holotype based on the male specimen.

**Table:** 

1	Propodus of pereopod 1 expanded on inferior margin	**2**
–	Propodus of pereopod 1 not expanded on inferior margin	**4**
2	Maxillipedal palp with three articles; endopod of uropod surpassing pleotelson	*** T. chinensis ***
–	Maxillipedal palp with four articles; endopod of uropod not surpassing pleotelson	**3**
3	Pereonite 1 as long as pereonite 2; incisor of mandible with two cusps; apical lobe of maxilla without seta; pleotelson with eight robust setae on posterior margin	*** T. spongillicola ***
–	Pereonite 1 1.7 times longer than pereonite 2; incisor of mandible with one cusp; apical lobe of maxilla with one seta; pleotelson with ten robust setae on posterior margin	***T.koreaensis* sp. n.**
4	Incisor of mandible with three cusps; maxillipedal palp with five articles	*** T. tonlesapensis ***
–	Incisor of mandible with one or two cusps; maxillipedal palp with three or four articles	**5**
5	Pereonite 1 longer than other pereonites, 2.0 times longer than pereonite 5; apex of pleotelson with truncated margin	*** T. lacustris ***
–	Pereonite 1 marginally longer or shorter than pereonite 5; apex of pleotelson with rounded margin	**6**
6	Pereonite 1 longer than pereonite 5; maxillipedal palp with three articles	*** T. caridophaga ***
–	Pereonite 1 shorter than pereonite 5; maxillipedal palp with four articles	*** T. picta ***

####### Tachaea koreaensis sp. n.

Taxon classificationAnimaliaIsopodaCorallanidae

http://zoobank.org/4FB4AD83-2912-448A-8734-19C4CAD443D6

Figures 5 , 6 , 7

######## Material examined.

Holotype: non-ovigerous female (4.8 mm, NIBRIV0000554215); Buheungji reservoir, Yeongcheon-si, Gyeongsangbuk-do, South Korea; 35°55'19"N, 128°59'14"E; 18 April 2013; approximately 2 m; coll. K.-S. Sim; ectoparasites of *Macrobrachiumnipponense*. Paratype: non-ovigerous female (4.3 mm, NIBRIV0000754063); Wolga reservoir, Wolga-ri, Gunnae-myeon, Jindo-gun, Jeollanam-do, South Korea; 34°29'36"N, 126°17'35"E; 23 September 2016; 1.4 m; using a landing net; coll. D.-H. Ahn, C. W. Lee, H.-M. Yang and J.-H. Song; ectoparasites of *Palaemonpaucidens*.

######## Etymology.

The specific name ‘*koreaensis*’ is derived from the name of the nation from which the specimens were collected.

######## Diagnosis.

Pereopods 1–3 propodus expanded with serrations on inferior margins. Pereopods 4–7 propodus with serrations on inferodistal margins. Mandible incisor with one cusp. Maxilla apical lobe with one seta. Maxillipedal palp with four articles. Pleotelson with ten robust setae on posterior margin.

**Figure 5.**
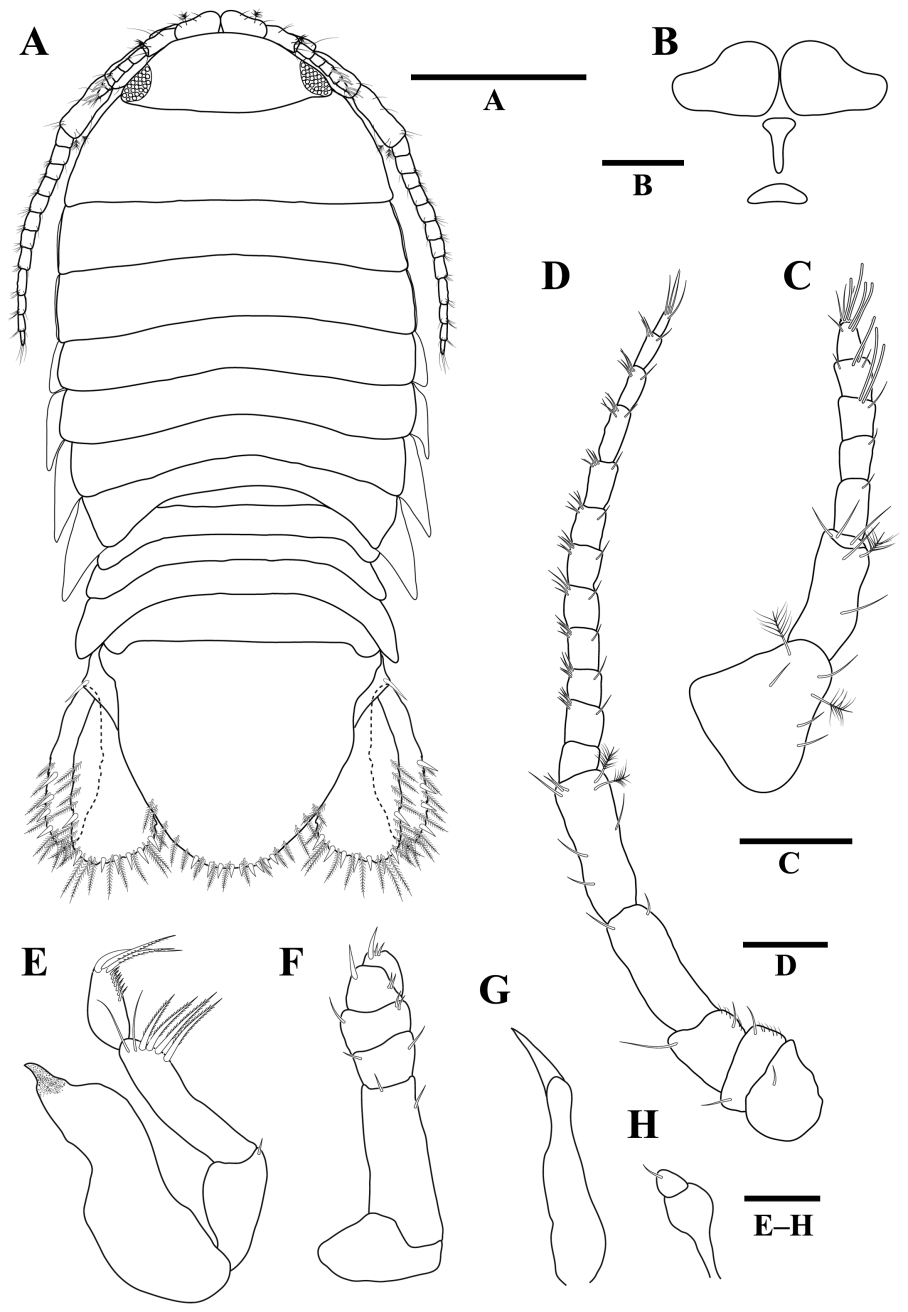
*Tachaeakoreaensis* sp. n., female holotype. **A** body, dorsal view **B** ventral view of bases of antennula, frontal lamina, and clypeus **C** antennula **D** antenna **E** mandible **F** maxilliped **G** maxillula **H** maxilla. Scale bars: 1 mm (**A**), 0.5 mm (**B**), 0.2 mm (**C, D**), 0.1 mm (**E–H**).

######## Description

**(non-ovigerous female, holotype).***Body* (Figure 5A) 2.2 times as long as greatest width. Colour yellowish; chromatophores on all somites, including both antennae and uropods. *Cephalon* (Figure 5A) 2.6 times wider than medial length. *Eyes* (Figure 5A) with 26–28 ommatidia. *Pereonite 1* (Figure 5A) longest, 1.7 times as long as pereonite 2 and 4, 1.4 times as long as pereonite 3, 1.8 times as long as pereonite 5, 2.1 times as long as pereonite 6, 2.8 times as long as pereonite 7.

*Frontal lamina* (Figure 5B) elongate, narrow; clypeus short and broad.

*Antennula* (Figure 5C) peduncle article 1 triangular, 1.1 times as long as wide, with four setae and two penicillate setae; article 2 0.7 times as long as article 1, with two setae and one penicillate seta; article 3 minute, 0.2 times as long as article 2, with three setae distally; flagellum with seven articles, articles 3–6 with 2 aesthetascs, articles 6 and 7 minute. *Antenna* (Figure 5D) peduncle article 1 0.8 times as long as wide, with one seta; article 2 shortest, with two setae; article 3 2.0 times as long as article 2, with two setae; article 4 with two setae; article 5 with six setae and two penicillate setae; articles 4 and 5 similar length; flagellum with 12 articles, each articles with six setae.

*Mandible* (Figure 5E) palp article 2 with four serrate setae and two setae; article 3 with seven short serrate setae, three serrate setae. Lacinia mobilis and molar process absent; incisor monocuspid. *Maxillula* (Figure 5G) lateral lobe forming single large curved spine. *Maxilla* (Figure 5H) short, apical lobe with one seta. *Maxilliped* (Figure 5F) without endite; palp with four articles, narrow, 4.4 times as long as wide.

**Figure 6.**
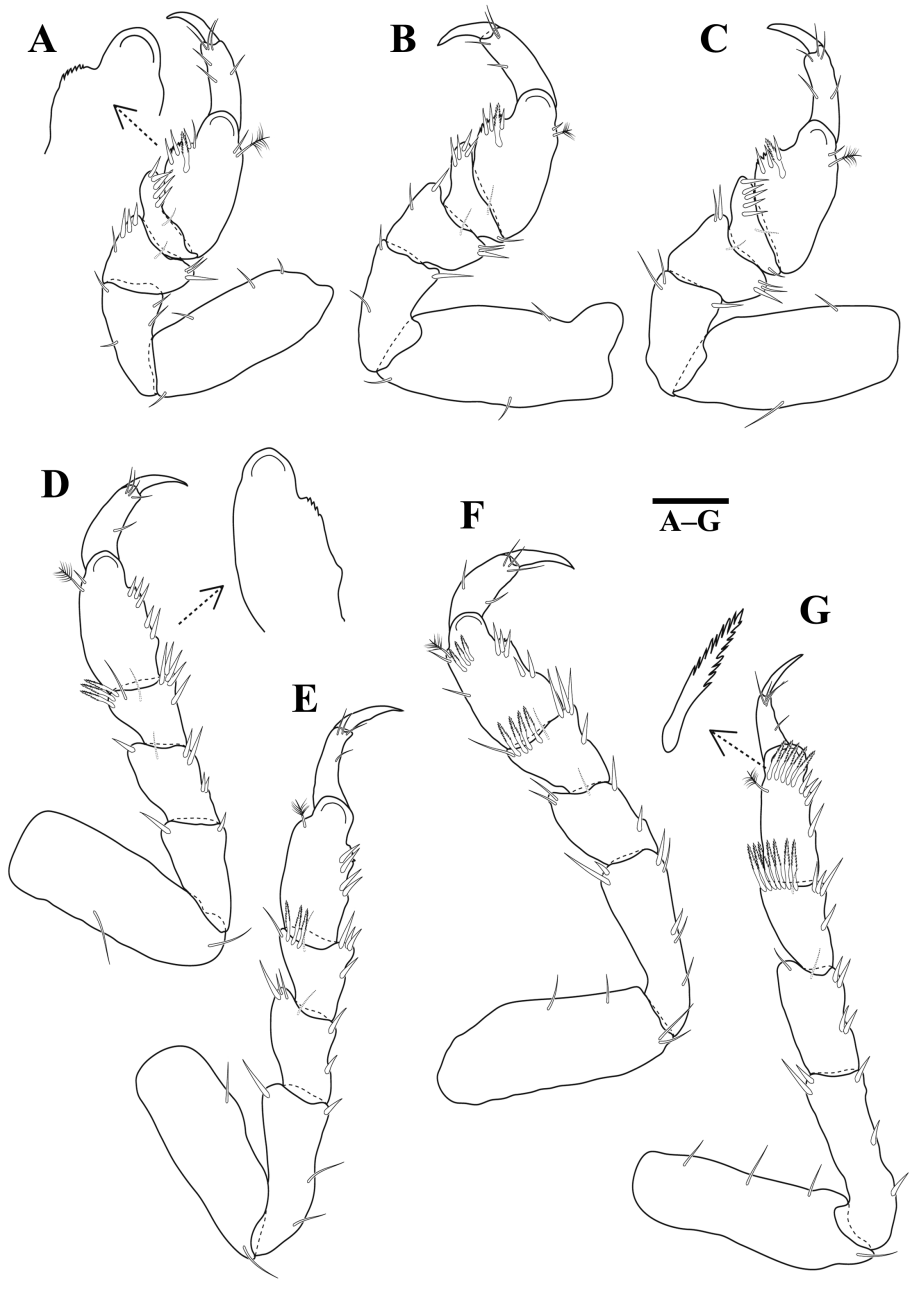
*Tachaeakoreaensis* sp. n., female holotype. **A** pereopod 1 **B** pereopod 2 **C** pereopod 3 **D** pereopod 4 **E** pereopod 5 **F** pereopod 6 **G** pereopod 7. Scale bars: 0.2 mm (**A–G**).

*Pereopods* 1–3 (Figure 6A–C) similar, propodus inferior margins expanded with serrations. Pereopod 1 basis 2.7 times as long as greatest width, superior margin with three setae, inferodistal margin with one seta; ischium 0.6 times as long as basis, 1.8 times as long as wide, superior margin with two setae, inferior margin with two setae; merus 0.6 times as long as ischium, 0.8 times as long as wide, superodistal margin with two setae and one robust setae, inferodistal margin with three robust setae, inferior margin with one seta; carpus shortest, 0.4 times as long as merus, 0.3 times as long as wide, superodistal margin without setae, inferodistal margin with five robust setae; propodus 1.2 times as long as ischium, 1.7 times as long as wide, superior margin with one seta and one penicillate seta, inferior margin with three robust setae, one comb seta and one seta; dactylus 0.5 times as long as propodus. Pereopods 4–7 (Figure 6D–G) similar, propodus inferodistal margins with serrations. Pereopods 4 and 5 carpi with three comb setae; pereopod 6 carpus with four comb setae, propodus with three comb setae; pereopod 7 carpus with seven comb setae, propodus with five comb setae.

**Figure 7.**
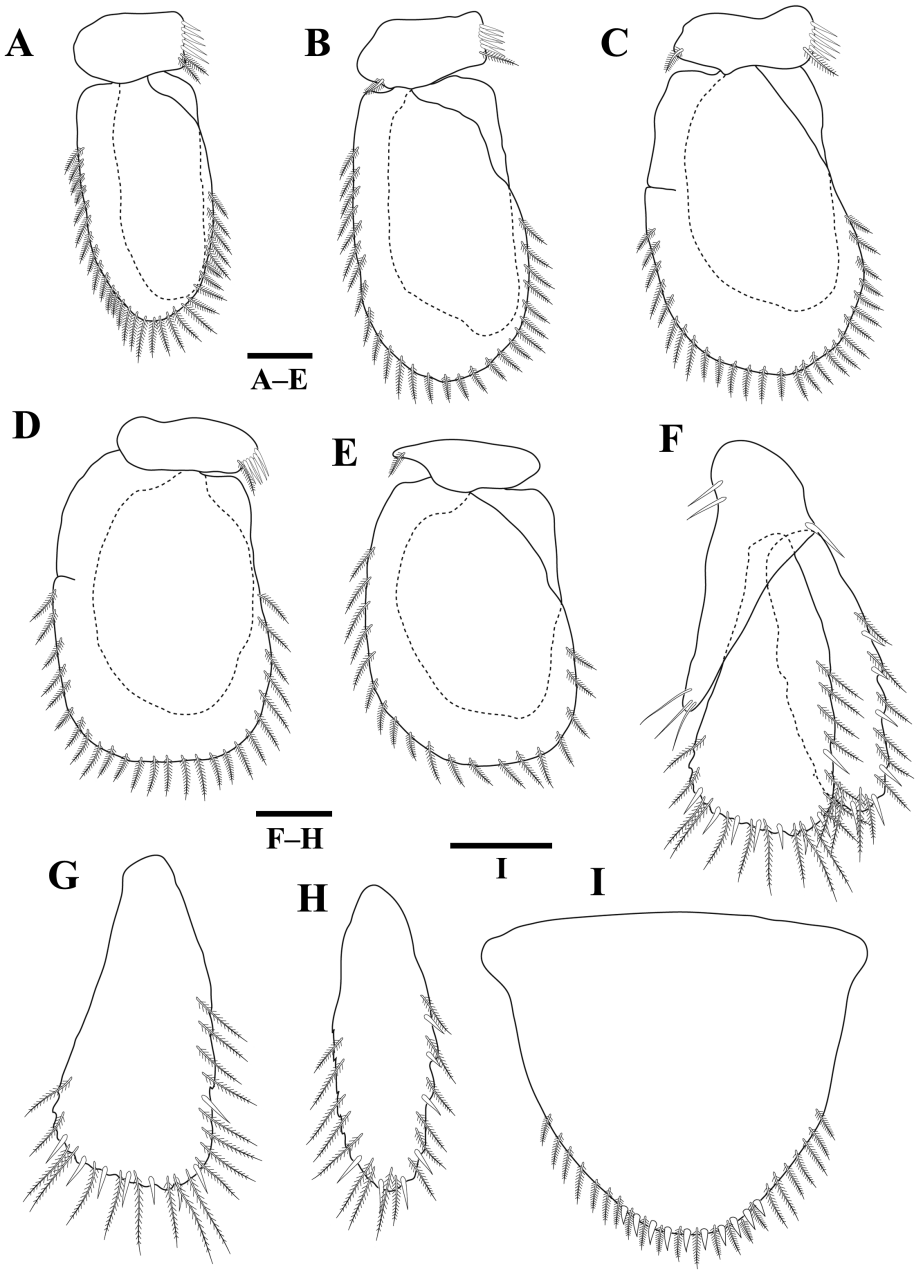
*Tachaeakoreaensis* sp. n., female holotype. **A** pleopod 1 **B** pleopod 2 **C** pleopod 3 **D** pleopod 4 **E** pleopod 5 **F** uropod **G** uropod endopod **H** uropod exopod **I** pleotelson. Scale bars: 0.2 mm (**A–H**), 0.4 mm (**I**).

*Pleopods* 1–5 (Figure 7A–E) similar; exopod broader, longer than endopod, with plumose setae; endopod naked. Pleopods 1–4 peduncle wider than long, with 5–6 coupling spines and 1–2 plumose setae. Pleopod 5 peduncle without coupling spines. Uropod (Figure 7F) rami reaching pleotelson; peduncle distal margin with three setae, proximal margin with two setae, lateral margin with one seta; endopod (Figures 5A, 7G) not surpassing pleotelson, 2.1 times as long as greatest width, lateral margin with one robust setae and plumose setae, mesial margin truncate, with seven robust setae and plumose setae; exopod (Figures 5A, 7H) not extending to end of endopod, 3.1 times as long as greatest width, lateral margin with four robust setae and plumose setae; mesiodistal margin with three robust setae and plumose setae. Pleotelson (Figure 7I) 0.8 times as long as anterior width; posterior margin rounded, with ten robust setae and numerous plumose setae.

######## Remarks.

*Tachaeakoreaensis* sp. n. is distinguished from other known species of *Tachaea* by the following combination of characters: 1) the inferior margins of the propodus of pereopods 1–3 is expanded with serrations, 2) the inferodistal margins of the propodus of pereopods 4–7 with serrations, 3) the apical lobe of the maxilla with one seta, and 4) the posterior margin of the pleotelson with ten robust setae.

*Tachaeakoreaensis* sp. n. is most similar to *T.spongillicola* from India, but it can be distinguished from the latter by the following characters: the ratio of pereonite 1 to pereonite 2, the number of cusps on the mandible, the presence or absence of setae on the apical lobe of the maxilla, and the number of robust setae on the posterior margin of the pleotelson. In *T.spongillicola*, the pereonite 1 is as long as the pereonite 2, the incisor of the mandible with two cusps, the apical lobe of the maxilla without seta, and the posterior margin of the pleotelson with eight robust setae. In comparison, in the new species, the pereonite 1 is 1.7 times as long as the pereonite 2, the incisor of the mandible with one cusp, the apical lobe of the maxilla with one seta, and the posterior margin of the pleotelson with ten robust setae.

######## Distribution.

Jeollanam-do and Gyeongsangbuk-do (South Korea).

## Supplementary Material

XML Treatment for Gnathia

XML Treatment for Gnathia koreana

XML Treatment for Tachaea

XML Treatment for Tachaea koreaensis
